# Change in the Torsional Stiffness of Rectangular Profiles under Bending Stress

**DOI:** 10.3390/ma15072567

**Published:** 2022-03-31

**Authors:** Krzysztof Macikowski, Bogdan Warda, Grzegorz Mitukiewicz, Zlatina Dimitrova, Damian Batory

**Affiliations:** 1 Department of Vehicles and Fundamentals of Machine Design, Lodz University of Technology, Stefanowskiego 1/15, 90-537 Lodz, Poland; bogdan.warda@p.lodz.pl (B.W.); grzegorz.mitukiewicz@p.lodz.pl (G.M.); damian.batory@p.lodz.pl (D.B.); 2 PSA Groupe, 78-140 Vélizy-Villacoublay, France; zlatina.dimitrova@mpsa.com

**Keywords:** rectangular profile, torsional stiffness, stiffness increase, research, finite element method

## Abstract

This article presents the results of research on the change in torsional stiffness of two rectangular profiles, arranged one on top of the other, which were permanently connected at their ends. The flat bars were expanded in the middle of their active length. The test involved determining the increase in the stiffness of a twisted test set before and after expanding. The authors present an analysis of the structure load and compare the results of tests carried out using analytical (for selected cases), numerical and experimental methods, obtaining satisfactory compliance. The analytical calculations included the influence of limited deplanation in the areas of the profile’s restraint. The ANSYS package software was used for calculations with the Finite Element Method. A change in the stiffness increase index at torsion was determined. The obtained results showed that expanding the test sets in their middle causes an increase in torsional stiffness, which is strongly dependent on the design parameters such as bending deflection, torsion angle and dimensions of the cross-section of the flat bar in the package.

## 1. Introduction

The torsion of rectangular profiles is a load state in which the profiles are affected by the torsional moment in their cross-section plane. If the bars are restrained, their condition is described as restrained torsion. Torsional stiffness is the measure of the profiles’ resistance to torsion and it depends on the material’s mechanical characteristics and geometrical dimensions of the twisted profile [1].

The results of torsion tests are applied in many areas. In the automotive sector, they help design, e.g., torsion bars of variable stiffness. In civil engineering, they are used in the load-bearing systems of buildings [2,3,4,5,6,7,8] and other structures [9].

Ribeiro and Silveira [10] investigated the influence of changes in torsional stiffness by changing the distance between a car stabiliser’s sleeves on the car body tilt while driving along an arch. The researchers determined that by increasing the test span from 100 mm to 300 mm, the tilt can be reduced from 2.76° to 2.65°. Without modifying the other structural parameters of the bar, they changed its stiffness from 7.87 kN/m to 9.35 kN/m.

Owing to the use of a hydraulic BMW Dynamic Drive hydraulic mechanism [11,12] which changes the torsional moment load of the middle part of the cross stabiliser (by counter-moment generation), the body tilt can be controlled depending on the vehicle motion conditions. For a vehicle that moves along a 40 m radius arch, it was reduced from ca. 3° to ca. 0.5° (at the lateral acceleration of 5 m/s^2^).

Contrary to the BMW hydraulic system, Buma [13] used an electrical mechanism in the central part of the bar, connected with a toothed gear that enables reaching high torque values. The torque rotates the torsional bar in the opposite direction; the bar consists of two coupled parts. The test car body’s tilt was reduced from ca. 2.1° to 1° at the lateral acceleration of 5 m/s^2^.

According to the results obtained by Doody [14] and Husen and Naniwadekar [15,16], the use of a metal sleeve (instead of a rubber one) for mounting the stabiliser increases its stiffness (including torsional stiffness of the central part) by 35% and 37%, respectively.

Shokry [17] investigated the strength of a rectangular beam made from polyester composite reinforced with glass fibre. The addition of nanoparticles was aimed at increasing the sample’s bending strength. Other researchers also focused on the issue of bent profiles used for car leaf springs [18,19].

The results of experimental tests on reinforcing narrow beams with carbon fiber-reinforced laminates were reported by Bakalarz et al. [20]. The authors obtained 11% and 7% increase in the global modulus of elasticity in the bending and stiffness coefficients, respectively.

Wan and Jung [2,21,22] examined an LSB (LiteSteel beam) with a C-shaped cross-section, used in floor systems as ground beams or supporting beams, whose centre of gravity does not overlap with the shear centre. The results revealed that the torsional moment rises as the shear centre eccentricity increases, which leads to a significant decrease in the beams’ resistance to the bending moment. Other researchers who carried out tests on beams with similar shapes obtained parallel results [23,24,25].

Structures used in everyday life are often exposed to complex states of stress. Simultaneous twisting and bending represent one of the most challenging cases. The literature review did not reveal any examples of two flat bars simultaneously subjected to such loads. In the presented paper, the numerical models of torsional stiffness of rectangular profiles under bending stress were determined. Additionally, the laboratory torsional tests of various sets of profiles with different cross-section shapes were conducted for the validation of the developed numerical models. The comparison of the results revealed good agreement between the registered numerical and experimental data, giving an opportunity of optimization of the profile shape and stiffening method of the analyzed sets in terms of achieving large changes in torsional stiffness with slight bending of the profiles.

### 1.1. Analysis of a Unexpanded Test Package

The presence of the cross-section limited deplanation results in auxiliary normal (tensile) stress occurring in addition to static (torsional) [26,27,28,29,30] stress in the profiles, contrary to pure torsion. This kind of torsion is called flexural torsion, and the total moment M_s_ necessary to twist the test set is a sum of two components: M_t,s_ and M_t,w_. The first corresponds to pure torsion (Saint Venant), while the other corresponds to flexural torsion moment.

M_s_ = M_t,s_ + M_t,w_ (1)

A literature analysis [31,32,33,34,35] reveals that the M_t,s_ and M_t,w_ shares change in a non-linear way along the calculated element. The first ones have a constant value, while the latter ones decay rapidly as the distance to the fixing points increases. The flexural torsion moment M_t,w_ depends on several factors:

- Dimensions of the cross-section (B and H)—the thinner and broader the flat bars, the more significant the share of M_t,w_ is in the total M_s_;
- Length (L)—the shorter the flat bars, the greater the share of M_t,w_ is in the total M_s_;
- Distances between the flat bars—the greater the length (to the cross-section’s shorter edge) between the flat bars, the more significant the share of M_t,w_ is in the total M_s_;
- Ratio of Young’s modulus (E; of longitudinal elasticity) to Kirchoff modulus (G; of volume elasticity)—an increase in the E and reduction of G values contribute to a higher share of the M_t,w_ limited warping moment in the M_s_ profile’s total torsional moment.

The outcomes of previous research [26,31] indicate that the torsional strength of open-section thin-wall profiles increases when torsional moments are taken over not only by unrestrained torsion but also by the forces warping the flanges in different directions, i.e., by flexural torsion stress. In other words, an increase in the M_t,w_ share in the total M_s_ makes a structure stiffer. In order for the strengthening effect to occur, the following two prerequisites have to be fulfilled:

- The cross-section shall be composed of at least two parallel flanges or at least three non-parallel walls that do not intersect in a single point;
- The fixing shall enable transferring the profile bending moments and shearing forces onto the support.

### 1.2. Analysis of an Expanded Test Package

As a result of the profiles’ expanding, additional bending stress occurs in the middle of the active length. The stress distribution changes, and so do the shares of M_t,s_ and M_t,w_ in the M_s_ transferred torsional moment. It can be evidence of the change in the torsional stiffness under expansion (bending stress).

The available literature misses examples of calculations for expanded flat bars. An analogy can be found in construction, where calculations for a twisted I-section present a similar case. If the analogy is used, the web thickness shall be assumed as zero. Unfortunately, the analogy can be applied only to flat bars whose shape did not change under expansion, i.e., distant from one another (e.g., for unexpanded profiles’ calculations). A sample calculation for I-sections is presented in EUROCODE 3 [34]. The method consists in the determination of a substitute warping moment called bimoment. To that end, the bending center shall be identified, and warping inertia determined.

The flexural torsion phenomenon and its influence on the torsional resistance of I-sections was explicitly presented in the SCI study [32], where the torsional stiffness coefficient was determined, along with its change depending on the beam length. Computational procedures were also presented for load cases common in engineering.

The literature hardly provides other examples to gain knowledge for a more detailed analysis of profiles under bending stress. There is no information about expanding arched beams. Moreover, there is a lack of information on bending or twisting elements with initial stress (analogy to stress that occurs when the profiles are expanded).

To summarize, it shall be concluded that it is hard to twist expanded bars. An approximate solution can be obtained in the best-case scenario, requiring long-lasting and labour-consuming analytical efforts.

This study includes experiments and simulation tests on two flat bars arranged one onto another, having a fixed length and variable cross-section dimensions. The flat bars were durably connected at the ends and expanded in the middle of their active length, ranging from 0 to 30 mm, and then twisted. The results of the tests can be applied in a design of a vehicle stabiliser of variable stiffness [36].

## 2. Materials and Methods

The tests were carried out on a package composed of two rectangular profiles connected permanently at the ends. The profiles were expanded in the middle of their active length and then twisted. The influence of bending on the change in test package torsional stiffness was analyzed.

**Figure 1:**
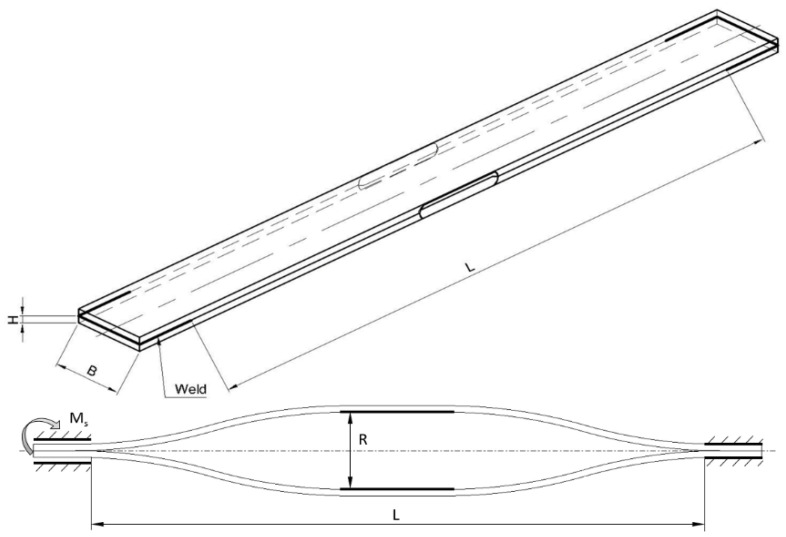
Schematic diagram of a test set (unexpanded on the top and expanded on the bottom).

The test set (Figure 1), with the twisted active length L amounting to 528 mm, consisted of one pair (two pieces) of flat bars, with the possible extension of the package with successive pairs. The tests were planned for 51CrV4 spring steel plate packages, 3 mm and 6 mm thick (H) and 30 mm to 60 mm broad (B). With regard to the mechanical resistance, including but not limited to thicker (higher) test sets, the maximum expansion (R) was limited to 30 mm. The maximum torsion value (φ) was assumed to amount to 20°. The assumed values result from an analysis of stabilizer bars used in typical passenger vehicles. An occurring space limitation and required resistance for twist (a torque needed to twist) in a middle part of the bar cause natural constraint for using shapes not exceeding the given values.

A load analysis enables the determination of two characteristic states: torsion of unexpanded profiles and torsion of expanded profiles. It was determined that the test package was exposed to the restrained torsion phenomenon and that the deplanation capacity of the flat bars in the restraint areas was limited. Moreover, the profiles were identified to be subject to a complex stress state that depends on the analysed form of the test set (unexpanded and twisted or expanded and twisted). The following types of stress were distinguished:

- Shear (tangential) stress originating from pure torsion;
- Tensile stress generated as a result of the profiles’ limited warping in the fixing areas;
- Bending stress along the longer edge of the cross-section) which occurs under tensile stress and shear forces (originating from restrained warping of the cross-sections);
- Bending stress along the shorter edge of the cross-section (resulting from the profiles’ bending).

### 2.1. Analytical Study

Analytical calculations of an unexpanded test package were carried out based on the material strength knowledge, including but not limited to thin-walled bars. The course of the calculations is not presented in this paper. Further sections include the obtained results that are compared with the results of numerical simulations and experiments.

### 2.2. Numerical Study

**Figure 2:**
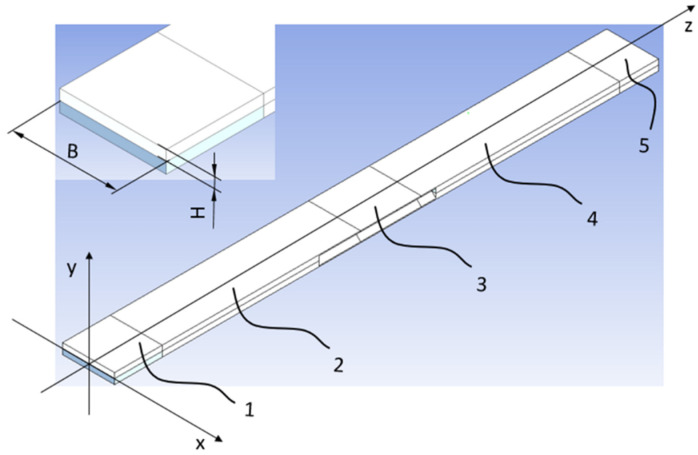
Numerical model. Successive sections are marked with digits from (1) to (5).

A simulation using the Finite Element Method was performed in the ANSYS environment [37,38]. The computational model’s structure reflected the real one in the restraining and loading method. Solid elements were used. This way, the edge folds used for mounting the bending mechanism were taken into consideration. Every profile was divided into five sections (Figure 2). The outer sections (1) and (5) corresponded to durable restraint. The middle section (3) was expanded. The other two sections, (2) and (4), were free, and their strain during simulation resulted from the restraining method and working conditions of the rectangular bars in the test stand.

A permanent bond was used at the contact planes of the flat bars’ outer edges. It corresponded to the processing capabilities of the test sets’ preparation (i.e., welding of the six available edges—see Figure 1). All degrees of freedom were taken on the outer planes of section (5). Friction between the rectangular bars (on Section 2, Section 3 and Section 4) was taken into consideration. For the unexpanded sets, the coefficient of friction amounting to μ = 0.2 was applied. For the expanded sets, it was μ = 0. This way, the mutual penetration of the profiles during twisting was avoided.

The model’s discretization was carried out in the Ansys Meshing tool, using non-linear mechanics settings. The Multizone algorithm was applied. HEXA20 and WED15 type cubic elements were generated. The first one was a dominant type. A single profile’s thickness in the test set was adopted as the mesh size. The number of the nodes changed depending on the model size and ranged from ca. 20,000 to ca. 80,000. The number of elements ranged from ca. 3500 to ca. 15,000. The mesh quality was checked with Orthogonal Quality indices (ca. 0.7–0.9), Skewness (ca. 0.2–0.5) and Aspect Ratio (ca. 1.5–5). The obtained values, depending on the test model’s dimensions, were sufficient for the simulation. No inflation or local concentration of the mesh was used. Quadratic element order function was applied just as it was done by Jafari [39].

**Figure 3:**
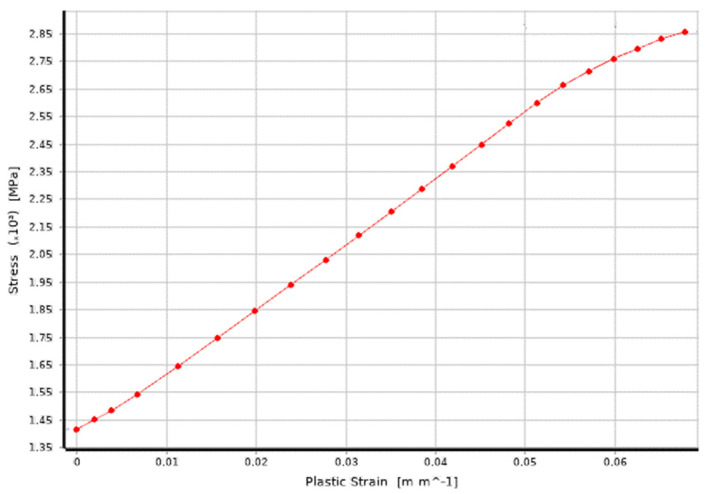
Non-linear model of a 6 mm thick material. The course of actual stress depends on the material deformation. The conventional yield point amounts to Re = 1417 MPa.

The analysis included a non-linear material model (Figure 3). Its parameters were obtained owing to a static tensile test. It was carried out for all tested flat bars’ thicknesses. The results were converted into real stress. Material stiffening under significant strain was also taken into account. The force, torque, displacement and rotation margins were assumed (from 0.05% to 0.1%). It enabled obtaining reproducible results with low sensitivity to the mesh size changes.

Bending and twisting were performed using the remote displacement function. It enabled to define necessary degrees of freedom, leaving the other ones non-defined. The displacement along axis *Y* (from 0 to ±15 mm) was applied to the inner planes of section (3). Twisting (by 20°) around axis *Z* and displacement equal 0 mm along axis *X* and *Y* was applied to the outer planes of section (1)—Figure 2.

**Figure 4:**
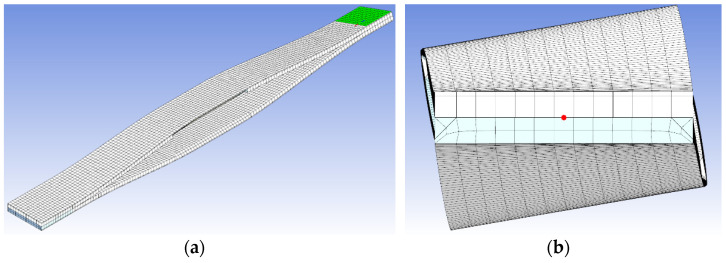
(**a**): test set expanded by 30 mm. (**b**): test set expanded by 30 mm and twisted by 20°.

The simulation was performed in two stages using the remote displacement function (Figure 4). In the first stage, the profiles were expanded, while in the second one they were twisted. The reactive moment was measured on the outer planes of section (1). In a purpose of strength control the reduced stress was checked.

The prepared model helped to perform numerical tests in the whole planned range. The tested profiles were 3 mm and 6 mm thick and 30 mm to 60 mm broad. They were expanded within the 0–30 mm range with a 5 mm stroke. The maximum twist amounted to 20°. The results obtained for unexpanded sets were compared with the results of analytical calculations. They were characterised by high convergence, ranging from −3% to +5.5%. It was then assumed that the simulation had been correctly prepared, and the results obtained for the expanded test packages were correct and confirmed by the experiment results.

### 2.3. Experimental Study

**Figure 5:**
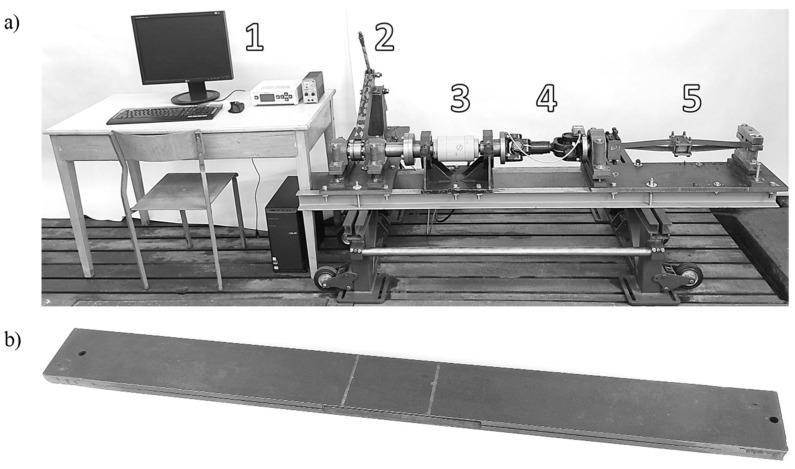
(**a**) Test stand. (**b**) Test set 6 × 60.

The developed test stand enabled obtaining reproducible measurement results at a short duration of a single set’s test. It consists of five essential assemblies, shown in Figure 5a.

A computer set (1) enables data saving and processing. The twisting of the test sets starts at the drive assembly (2), where it is forced with a mechanical lever and a screw-nut transmission powered by an electric motor. It ensures continuous moment and torsion measurement. The rotational motion is transferred by the assemblies of the torque meter (3) and a double-joint shaft (4) to the test set (5), where the tested profile packages are installed; the torque meter and double-joint shaft assemblies are connected in series.

The test sets are mounted on supports. One end of each test set is permanently connected, while the other (connected with the double-joint shaft) enables rotation along the profile’s lengthwise axis and compensation of its length change resulting from expansion and twisting. A tilt sensor is also installed there. The sets are expanded with spacer wedges, from 5 mm to 30 mm thick, with a 5 mm stroke. Pressure plates press them from the outside.

The sensors used during the tests included:

- Torque meters (MI130, MI500 and MI1000, Poznan, Poland) with a measurement range from 0 to 1000 Nm, measurement accuracy 0.99%;
- Posital Fraba ACS-080-2-SV20-HE2-2W inclinometers (HeerlenHeerlen, The Netherlands) with two measurement axes (each with a ±90° range), measurement accuracy 1.40%.

Additional calibration of torque meters was conducted by applying the particular static force to an arm of known length.

The test sets consist of two flat bars of the same length. In the central part of each flat bar, there are two folds of the inner edges that enable their use in subsequent tests. The profiles were made of 50 HF spring steel plate and connected at the ends by welding. Then they were quenched to ca. 46–48 HRC and tempered. The profiles are shown in Figure 5b.

The system operation was checked after installing the test packages on the test stand. Test twisting was performed, and the operation of the limit switches and tension of fixing bolts were checked. The repeatability of the test was verified by preliminary torque comparison for minimum and maximum profiles twist. The preliminary twisting was repeated three times for each analyzed profile set to eliminate the clearance. Subsequently the consistency was verified by torque curves comparison. After the preliminary inspection, the actual measurement was carried out. The profiles were twisted from 0° to 22°, while recording the torque and twist angle. After reaching the desired twist angle, the system switched off automatically and saved the data. The unloading measurement was performed in the same way. A reduction in the maximum recorded torsion was observed as the stiffness of the test sets increased. The changing stiffness forced the use of three torque meter types, adapting the measurement range in this way.

The test results were processed. Noise and interferences were removed, and the measurement range adapted (from 0° to 20°). Diagrams of the torque change depending on the torsion were developed for the data prepared this way. Trend lines were plotted for the obtained wavelengths. The best representation was obtained for the fourth-order polynomial. Based on these, the torsional moment was calculated and its results were used for determining the K stiffness increase index.

## 3. Results

### 3.1. Numerical Tests

**Figure 6:**
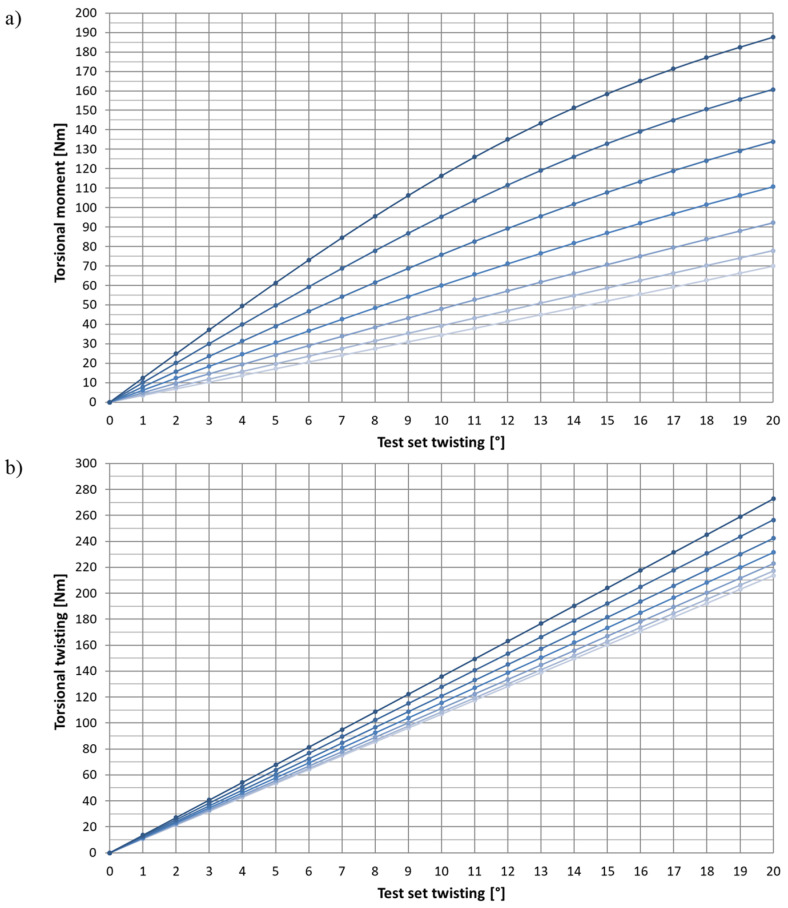
Change in the torsional moment of the 3 × 60 and 6 × 30 test sets during the numerical twisting test. The following expansions are marked with colors from the lightest to the darkest: 0, 5, 10, 15, 20, 25, and 30 mm. (**a**) 3 × 60 package. (**b**) 6 × 30 package.

A change in the moment depending on the test package twisting, and expansion was analyzed during the numerical tests for two groups—3 mm and 6 mm thick. The plotted diagrams were used for subsequent analyses. Sample diagrams (plotted for the cross-section extreme dimensions: H × B—3 × 60 and 6 × 30) are shown in Figure 6. The color intensity of each curve corresponds to successive expansions levels—from 0 mm (the lightest) to 30 mm (the darkest).

It was observed that a non-linear waveform characterized the packages made of thin and broad profiles (3 × 60). The more evident, the more expanded the test sets were. The diagrams (Figure 6a) reveal a decreasing moment gradient between successive torsion levels. The described effect confirms the growing share of the flexural moment M_t,w_ in the entire profile torsional moment M_s_. The observed bending of the curves in the plot reveals the non-linear dependence of M_t,w_ on the torsion.

The results of numerical tests are summarized in Table 1. The first column on the left includes the dimensions of a single profile cross-section dimensions (H × B; height (thickness) × breadth). The top row shows the successive expansion values from 0 (no expansion) to 30 mm. In order to facilitate understanding, each level is preceded by the R index. The entered values represent the torsional moment for a 20° twist.

In both analyzed thickness groups (3 mm and 6 mm), reproducible regularities can be observed. The torsional moment increases depending on the expansion. The lowest values occur for unexpanded test profiles, while the highest ones are achieved for the maximum expansion amounting to 30 mm.

The 3 mm thick sets are characterized by a measurement range from 30.4 Nm to 188.6 Nm. For the 6 mm thick packages, the values range from 214.4 Nm to 919.8 Nm.

The results of the numerical experiment helped to determine the profiles’ stiffening under bending. A per cent K stiffness increase index was developed according to formula (2) as a quotient of torsional moment read for expanded profiles M_s,R_ to torsional moment read for not expanded profiles M_s,0_:

K = [(M_s,R_/M_s,0_) × 100] − 100 [%] (2)

The index shows the value by which the torsional stiffness of the bar’s working part changed. For instance, a 10% increase means that the test set generates a 10% higher response moment than before the expansion. An increase by 100% signifies a two-fold increase in the torsional stiffness. The calculated values of the K index are summarised in Table 2, which shall be interpreted strictly in the same manner as Table 1.

In both analysed thickness groups (3 mm and 6 mm), repetitive regularities can be observed. The index value increases from 0% (for unexpanded profiles) to the maximum value (achieved for 30 mm expansion), and the growing breadth of the test packages contributed to higher stiffness gains.

Thin sets (3 mm) responded to expansion most sensitively. The maximum value of 171.3% was obtained for the 3 × 60 package, while the minimum of 77.0% was obtained for the 3 × 30 package. Thick sets (6 mm) were less susceptible to expansion. The maximum value amounting to 82.6% was obtained for the 6 × 60 package, while the minimum was 27.5% (for the 6 × 30 package).

Experiments confirmed the results of numerical simulations.

### 3.2. Experiments

The experiments covered the same test sets as the ones used in numerical simulation. The packages consisted of two profiles with a single flat bar thickness of 3 and 6 mm. The expansion was performed within a 0 mm to 30 mm range, with a 5 mm stroke.

Hysteresis between the profiles’ loading and unloading was observed during the tests. The hysteresis resulted from inner friction in the test package material [40] and flexibility of the test stand elements.

**Figure 7:**
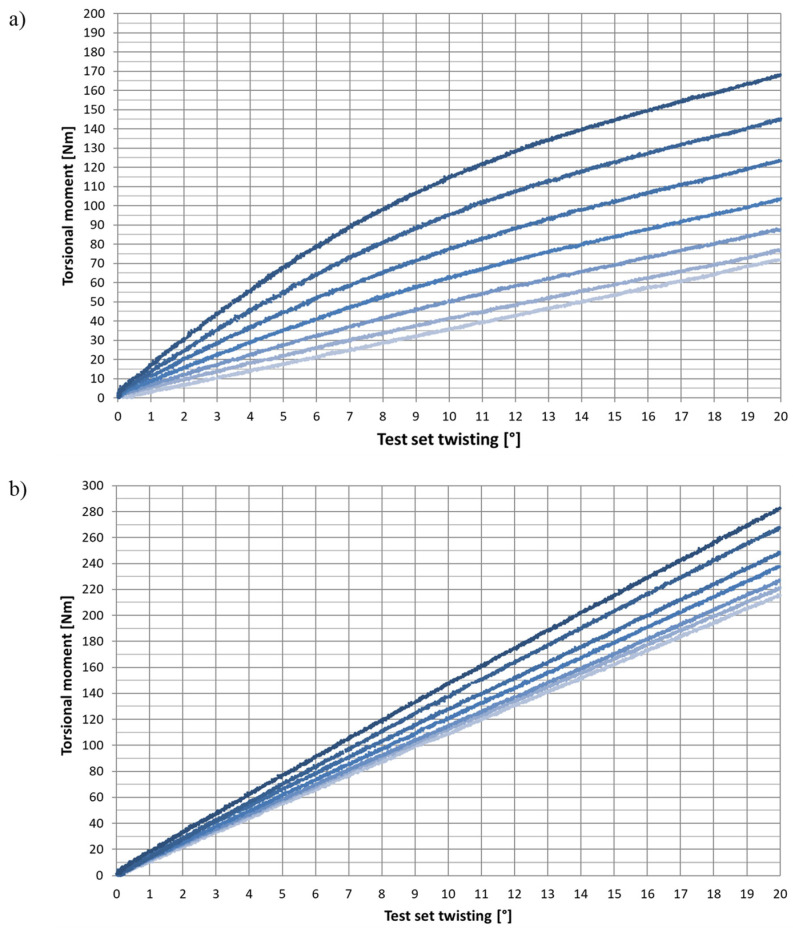
Experiment. Torsional moment (torque) change depending on the test set torsion. The following expansions are marked with colors, from the lightest to the darkest: 0, 5, 10, 15, 20, 25 and 30 mm. (**a**) 3 × 60 package (**b**) 6 × 30 package.

The results were processed to remove the noise and interferences. A diagram of the torque change during twisting was prepared for each test set. Only the curves obtained while twisting the profiles were used for their analysis. The comparison between the particular curves obtained for the same sample sets revealed that the coincidence was satisfactory (less than 0.5% for different twist values) and did not require more precise data processing. Sample waveforms for 3 × 60 and 6 × 30 sets are shown in Figure 7. The color intensity of each curve corresponds to successive expansion levels, from 0 mm (the lightest) to 30 mm (the darkest).

The waveforms of the unexpanded profiles were nearly linear. The expanded sets, similar to the results of the numerical experiment, were characterised by non-linearity. It was most evident for thin (3 mm), broad (mostly at 60 mm) and expanded (mostly at 30 mm) profiles. In each of these situations, the moment’s gradient between subsequent torsion levels revealed decreasing values. Trend lines were plotted for the obtained wavelengths. The best representation was obtained for the fourth-order polynomial. The functions enabled the moment calculation at a 20° twist of the test sets. The results are summarised in Table 3.

Similar to the numerical experiment, the torsional moment values were observed to increase with the test set’s thickness, breadth, and expansion. The lowest values in each group were read for narrow, unexpanded profiles. Analogically, the highest ones applied to broad profiles at maximum expansion.

The 3 mm thick sets are characterised by the measured moment range from 30.5 Nm to 168.0 Nm. For 6 mm test packages, it was from 215.8 Nm to 846.8 Nm.

The results of the experiment carried out in the test stand enabled the determination of the profile’s stiffening under bending. The per cent K stiffness increase index was calculated according to formula (1). The index values are summarised in Table 4.

Repetitive regularities can be observed in both analysed thickness groups (3 mm and 6 mm). The index value rises from 0% (for unexpanded profiles) to the maximum value (achieved for 30 mm expansions). The increasing breadth of the test packages causes higher stiffness gains.

The profiles responded most intensively to expansion in thin sets (3 mm). A maximum of 133.3% was achieved for the 3 × 60 mm package, while a minimum of 79.8% was achieved for the 3 × 30 package. Thick sets (6 mm) demonstrated lower susceptibility to expansion. A maximum of 69.3% was achieved for the 6 × 60 package, while a minimum of 29.7% was achieved for the 6 × 30 package.

## 4. Discussion

### 4.1. Accurancy Analysis

To provide good quality results discussion, it is helpful to know the measurement error. Simulation and experimental uncertainty were estimated.

#### 4.1.1. Experiment Test

The errors in experiments include:

1. A systematic error (δ_i_ of the inclinometer, δ_m_ of the torque meter and δ_ad_ of the measurement results proximation). It amounts to:
  δ_p_ = ±√ (δ_i_^2^ + δ_m_^2^ + δ_ad_^2^) = ±2.63%
2. A geometric error. It is affected by:
  - Position of distance elements—δ_ed_;
  - Initial twisting of the test set in the rotary support clamp—δ_sp_;
  - Test set installation in the test stand—δ_mp_;
  - Yest stand execution tolerances—δ_ks_;
  - Non-homogeneity of the material that the test sets are made of—δ_nm._
  The geometric error amounts to:
  δ_g_ = ±√ (δ_ed_^2^ + δ_sp_^2^ + δ_mp_^2^ + δ_ks_^2^ + δ_nm_^2^) = ±4.77%
3. The δ_bp_ random error was assumed as:
  δ_bp_ = ±5%

#### 4.1.2. Numerical Simulations

It is hard to estimate a numerical simulation error. It is affected by divergences between the experiment and simulations and FEM errors. Based on experience and professional literature [41], the following accuracy of the computational model was assumed:

δ_s_ = ±5%

#### 4.1.3. Accuracy of Measurements

The δ_e_ experimental method’s accuracy was determined as:

δ_e_ = ±(δ_p_ + δ_g_ + δ_bp_) = ±12.40%

Therefore, the maximum difference between the results obtained on the test stand and the results of numerical simulations is:

δ_c_ = ±(δ_e_ + δ_s_) = ±17.40%

### 4.2. Analysis of the Moment Twisting the Unexpanded Test Sets

Unexpanded test sets in each measurement series generated the lowest twisting moment. It represents the reference value necessary to determine the per cent K stiffening increase index for flat bars. Table 5 summarises the results of three test methods obtained for the profiles twisted by 20°. The values were similar. The knowledge of the differences will help determine the accuracy of the tests.

**Figure 8:**
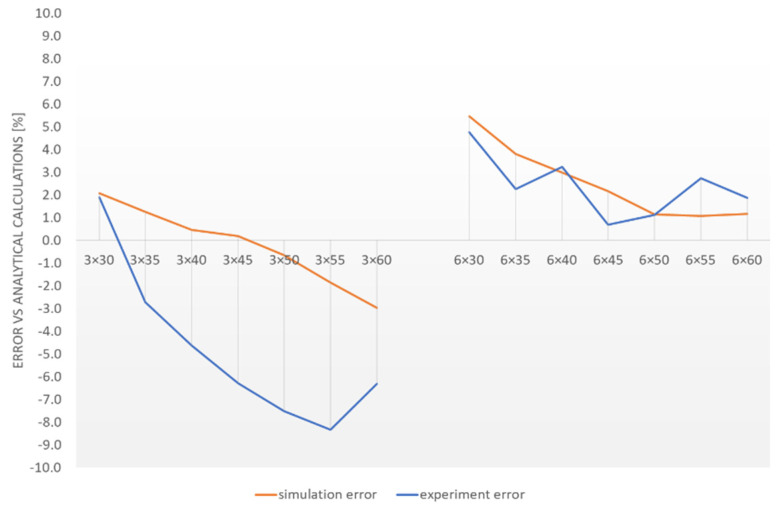
Per cent error of the numerical and experimental methods versus the analytical method.

Based on Table 5, the numerical and experimental method error versus the analytical method were determined. The obtained values were summarised as a diagram in Figure 8. The values lower than one (ordinate axis) meant higher torque values than those suggested by analytical calculations and vice versa for the lower ones.

An evident declining trend characterised all waveforms. Its occurrence can be explained by material loss in the central section of the flat bars, occurring when the edges are milled to mount the expanding mechanism. Residual stress in the material (occurring during heat treatment) can also contribute to the observed curves’ waveform; they are larger for broader profiles.

Similar error values characterised the 6 mm thick sets in each test. They ranged from +0.7% to +5.5%. The differences between the experimental and numerical methods revealed negligibly low differences (max. 1.6% for the 6 × 55 package).

Divergences can be observed for the 3 mm thick packages. The highest error was observed during the experiment (3 × 55 package) and amounted to 8.3%. The comparison of the results obtained in the simulation and the experiment reveals significant differences. A variable error ranging from 6.5% to 6.9% was observed between the 3 × 45, 3 × 50 and 3 × 60 packages. It can be concluded that test sets with low stiffness (3 mm) are more susceptible to the impact of the test stand execution inaccuracies and mounting errors. This was observed in the three presented tests.

**Figure 9:**
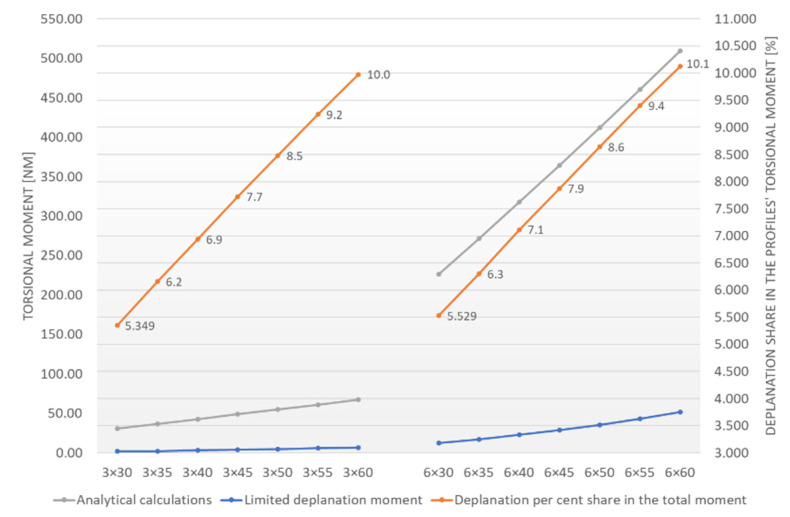
Limited deplanation share in the Ms total torsional moment affecting the unexpanded test sets.

The influence of limited deplanation on the entire torsional moment M_s_ was considered in the calculations. It’s percent share is marked in orange in Figure 9 (the right ordinate axis). Grey corresponds to the M_s_ torsional moment (the left ordinate axis), while blue corresponds to the restrained warping moment (the left ordinate axis).

The diagram analysis reveals that the limited deplanation moment increases with the test sets’ breadth. Its per cent shares take similar values, regardless of the thickness of the flat bars in the set. They range from 5.3% to 10.1%. The per cent differences between the corresponding values (e.g., 5.3% corresponds to 5.5% for the 3 × 60 and 6 × 30 sets) do not exceed 3.5%. This is helpful information for a designer who constructs a solution based on a similar principle. Instead of time-consuming calculations of limited deplanation, the result obtained for pure torsion can be increased by λ deplanation dimensionless value. The formula (3) can be used for this purpose:

M_s_ = M_t,s_∗[(100/(100 − λ)]  [Nm] (3)

### 4.3. Comparison of the Simulation and Experimental Results

#### 4.3.1. Torsional Moment Research

##### Non-Linearity of the Torsional Moment’s Wavelength

The tests were carried out to determine the stiffening after twisting the set by 20°. The influence of the non-linear waveform of the moment when a non-expanded set is twisted must not be neglected in order to understand the phenomenon comprehensively. For a nearly linear waveform (for the unexpanded package), it causes the variability of the K stiffness increase index.

**Figure 10:**
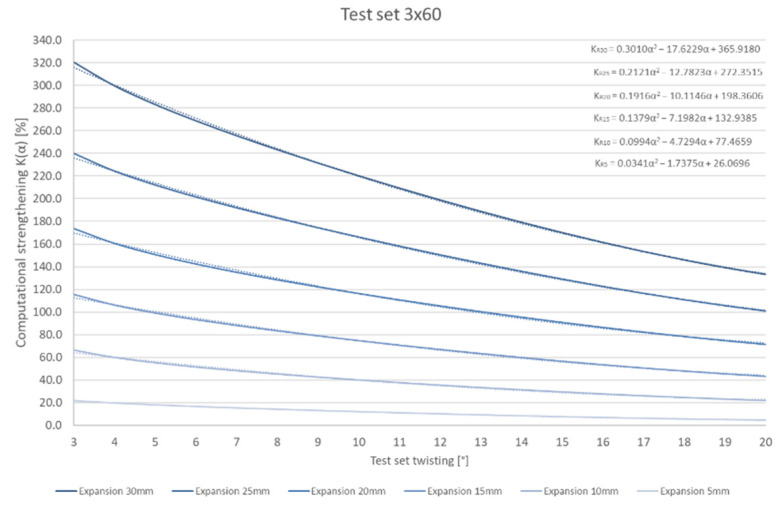
Variable value of the K stiffness increase index depending on the test set twisting.

An additional analysis of the K stiffness increase index change was performed for the 3 × 60 set (based on the experiment results). The scope of the study was limited to the torsion range from 3° to 20°. Interferences disturbing the observations occurred below the minimum value. The maximum strengthening for the most significant expansion (R = 30 mm) amounts to 320.3% (at twisting by φ = 3°), whereas for twisting by φ = 20°, it was only 133.3%. Similarly, though not as high, differences can be read for other expansions. The results are summarised in the diagram in Figure 10.

The analysis above suggests the possibility of changing the K stiffness increase index value by changing the twisting range. For the 3 × 60 package, the K index increased by 2.4 times.

##### Results of Torsional Moment Tests

The results obtained with the numerical and empirical methods were compared. An error versus the tests performed in the test stand was calculated and is summarised in Table 6. A three-colour scale was used for better understanding. The stronger colour indicates a higher per cent difference between the compared moments. Red (positive values) indicates a moment lower than the one obtained in the experiment, while blue indicates the opposite. White stands for the same value in both measurement methods.

An analysis of the error distribution helps us to notice that the numerical model stiffening trend dominates. As expansion increases, red fades and turns into blue. This means that the simulation model becomes stiffer earlier than the experimental one.

The differences exceeding 10% were obtained for the broadest set (3 × 60) at two measurement points—at 25 mm (11.4%) and 30 mm (12.3%) expansion. In the group of packages made of 6 mm thick steel plate, the most significant deviation (for the 6 × 60 test set) amounted to 8.6%. The quoted values do not exceed the calculated measurement difference (±17.4%).

The differences between the experiment and simulation results are caused by inaccurate test stand execution, stiffness, the technology of making the test sets, non-homogenous material structure, and divergent numerical simulations versus real conditions. High deviation values observed for the broadest packages may result from a simplified method of applying the nodes in the fixing areas (FEM simulation). The area of the mounting elements in the test stand was wavy, whereas in the numerical method, it was a plane. This could contribute to the occurrence of micro-motions or local material upsetting during the test, which reduced the value of the read torsional moment in real conditions.

The mean relative error amounted to:

- 4.1% for the test sets made of 3 mm steel plate,
- 2.2% for the test sets made of 6 mm steel plate,
- 3.1%—error for all tests.

The mean result for the relative error lower than 5% confirms that the FEM model was correctly prepared. None of the calculated deviations exceed the determined maximum measurement difference of 17.4%.

#### 4.3.2. K Stiffness Increase Index

The K stiffness increase index informs about the quantitative increase in the torsional moment affecting the test set under expansion. The torque measured for non-expanded profiles is the reference value, hence in this case, the coefficient value is always 0%. It was calculated for the measurements made with the numerical and experimental method.

Two factors were hampering the analysis of the K stiffness increase index. Firstly, the moments read for each measurement method had different values. It resulted from measurement differences (mentioned in Section 4.1). Secondly, the comparison of values suffering from deviations independent of one another causes a risk of their overlapping and consequently strengthening or weakening.

Due to the above-mentioned factors, it was decided to not compare numerical and experimental K index results. Such results would not be a reliable source of knowledge about achievable strengthening. Still, they can be used to estimate the strengthening achievable for the particular test set.

## 5. Conclusions

This paper is devoted to the study of change in the torsional stiffness of expanded rectangular profiles connected permanently on both ends. Two bending stress states were analysed: first, twisted flat bars, and second, expanded and twisted flat bars. The first state was tested with analytical, numerical, and experimental methods; the other was based on FEM simulation and doing an experiment at the test stand. Moreover, a measurement error was analysed, the share of limited deplanation in the total torsional moment investigated, and the stiffness increase index change during twisting determined.

The analysis of the study works leads to the following conclusions:

- An increase in the torsional stiffness of the flat bars depends on their cross-section, expansion, and angle of twist.
- An analysis of the structure load reveals that stiffening of the flat bars depends on the tensile stress resulting from cross sections’ limited deplanation in the fixing areas.
- The share of the torque resulting from restrained warping in the total torsional moment strongly depends on the breadth and takes similar values for the test sets made of 3 and 6 mm thick flat bars. It ranges from 5.3% to 10.1%, and the developed change diagram can be helpful for simplified calculations of similar structures.
- The comparison of the results revealed FEM error versus the experiment amounting in average to 3.1%, which is a satisfactory value.

Positive results obtained for test sets made of spring steel suggest that the use of modern composite materials would allow us to obtain a higher stiffness increase index.

According to the authors, the study results can be used for designing a car stabiliser that actively changes its stiffness. The decreasing torque gradient during twisting of the expanded working part can positively influence travelling comfort and safety, fulfilling the function of informing the driver about approaching the steerability limit.

## 6. Patents

Dmitrova Z, Kaszuba S, Macikowski K. Systeme Anti-Devers A Raideur Variable Comportant Des Series De Barres Qui S’ecartent Entre Elles. FR3 057813, 2018.

## Figures and Tables

**Table 1:** Results of numerical simulations. Torsional moment [Nm] for 20° twist, depending on the package’s cross-section dimensions (H × B) and the expansion R [mm].

Sample H × B [mm]	R0	R5	R10	R15	R20	R25	R30
**3 × 30**	30.4	31.0	33.2	36.8	41.3	47.2	53.8
**3 × 35**	36.4	37.5	40.9	46.4	53.2	62.1	71.9
**3 × 40**	42.6	44.3	49.3	57.2	66.9	79.1	92.6
**3 × 45**	48.7	51.6	58.4	69.1	81.9	97.8	114.6
**3 × 50**	55.3	59.2	68.2	82.1	98.3	117.8	137.7
**3 × 55**	62.3	67.3	78.7	95.9	115.6	138.3	161.2
**3 × 60**	69.5	75.7	89.8	110.7	134.1	161.2	188.6
**6 × 30**	214.4	217.2	222.9	231.6	242.4	256.5	273.3
**6 × 35**	261.5	265.0	273.9	287.6	304.3	326.2	351.4
**6 × 40**	308.4	314.6	327.7	347.7	372.3	404.4	440.9
**6 × 45**	356.7	366.4	384.8	413.0	447.4	492.3	543.2
**6 × 50**	407.4	420.2	445.2	483.4	529.9	590.2	658.3
**6 × 55**	455.6	476.4	509.3	559.4	620.2	698.8	787.0
**6 × 60**	503.7	529.1	571.9	635.5	712.3	810.7	919.8

**Table 2:** Numerical simulation. Summary of the percentage value of the K stiffness increase index, depending on the test package (H × B) and expansion R [mm].

Sample H × B [mm]	R0	R5	R10	R15	R20	R25	R30
**3 × 30**	0.0	1.9	9.3	21.1	35.8	55.1	77.0
**3 × 35**	0.0	2.9	12.4	27.5	46.2	70.4	97.4
**3 × 40**	0.0	4.0	15.7	34.3	57.0	85.8	117.3
**3 × 45**	0.0	5.8	19.8	41.8	68.1	100.6	135.1
**3 × 50**	0.0	7.1	23.3	48.4	77.7	113.0	149.1
**3 × 55**	0.0	8.1	26.3	54.0	85.5	122.0	158.8
**3 × 60**	0.0	9.0	29.2	59.2	92.9	131.9	171.3
**6 × 30**	0.0	1.3	4.0	8.0	13.0	19.6	27.5
**6 × 35**	0.0	1.4	4.8	10.0	16.4	24.8	34.4
**6 × 40**	0.0	2.0	6.2	12.7	20.7	31.1	43.0
**6 × 45**	0.0	2.7	7.9	15.8	25.4	38.0	52.3
**6 × 50**	0.0	3.1	9.3	18.6	30.1	44.9	61.6
**6 × 55**	0.0	4.5	11.8	22.8	36.1	53.4	72.7
**6 × 60**	0.0	5.1	13.5	26.2	41.4	61.0	82.6

**Table 3:** Results of the bench experiment. Torsional moment Ms [Nm] for 20° twist, depending on the package’s cross-section dimensions (H × B) and expansion R [mm].

Sample H × B [mm]	R0	R5	R10	R15	R20	R25	R30
**3 × 30**	30.5	32.2	34.5	37.9	42.7	48.4	54.8
**3 × 35**	37.9	39.9	43.6	49.4	56.5	64.4	73.5
**3 × 40**	44.9	46.9	52.4	59.9	68.9	80.7	92.9
**3 × 45**	52.1	55.2	61.5	70.7	82.8	97.6	112.4
**3 × 50**	59.4	63.0	71.1	82.9	97.7	115.4	133.5
**3 × 55**	66.7	71.1	80.5	94.7	111.8	131.3	152.8
**3 × 60**	72.0	75.5	87.8	103.2	123.4	144.7	168.0
**6 × 30**	215.8	220.5	226.1	237.3	245.9	268.0	279.9
**6 × 35**	265.4	270.5	279.2	292.8	311.2	330.0	353
**6 × 40**	307.7	318.8	336.1	358.4	373.6	407.5	441.0
**6 × 45**	362.0	377.5	399.8	426.5	446.2	494.2	524.3
**6 × 50**	407.6	418.0	453.7	487.7	508.1	577.9	636.2
**6 × 55**	448.3	472.8	506.5	553.6	584.9	674.7	753.3
**6 × 60**	500.2	521.2	572.5	624.8	664.6	764.4	846.8

**Table 4:** Results of the bench experiment. Summary of the percentage value of the K stiffness increase index, depending on the test package (H × B) and expansion R [mm].

Sample H × B [mm]	R0	R5	R10	R15	R20	R25	R30
**3 × 30**	0.0	5.6	13.2	24.6	40.2	58.8	79.8
**3 × 35**	0.0	5.2	15.1	30.2	49.2	69.8	93.8
**3 × 40**	0.0	4.5	16.8	33.4	53.5	79.9	107.0
**3 × 45**	0.0	6.0	18.1	35.6	58.9	87.3	115.8
**3 × 50**	0.0	6.1	19.8	39.5	64.5	94.3	124.7
**3 × 55**	0.0	6.6	20.6	42.0	67.6	96.9	129.1
**3 × 60**	0.0	4.9	21.9	43.3	71.4	101.0	133.3
**6 × 30**	0.0	2.2	4.8	9.9	14.0	24.2	29.7
**6 × 35**	0.0	1.9	5.2	10.3	17.3	24.3	33.0
**6 × 40**	0.0	3.6	9.2	16.5	21.4	32.4	43.3
**6 × 45**	0.0	4.3	10.4	17.8	23.3	36.5	44.8
**6 × 50**	0.0	2.6	11.3	19.7	24.7	41.8	56.1
**6 × 55**	0.0	5.5	13.0	23.5	30.5	50.5	68.0
**6 × 60**	0.0	4.2	14.5	24.9	32.9	52.8	69.3

**Table 5:** Summary of the results of calculating the torsional moment [Nm] of unexpanded test sets using analytical, numerical and experimental methods.

Sample H × B [mm]	Analytical Calculations	FEM Simulation	Experiment
**3 × 30**	31.0	30.4	30.5
**3 × 35**	36.9	36.4	37.9
**3 × 40**	42.8	42.6	44.9
**3 × 45**	48.8	48.7	52.1
**3 × 50**	54.9	55.3	59.4
**3 × 55**	61.1	62.3	66.7
**3 × 60**	67.5	69.5	72.0
**6 × 30**	226.1	214.4	215.8
**6 × 35**	271.4	261.5	265.4
**6 × 40**	317.6	308.4	307.7
**6 × 45**	364.5	356.7	362.0
**6 × 50**	412.1	407.4	407.6
**6 × 55**	460.5	455.6	448.3
**6 × 60**	509.5	503.7	500.2

**Table 6:** Summary of the results of calculating the torsional moment [Nm] of unexpanded test sets using analytical, numerical and experimental methods.

Sample H × B [mm]	R0	R5	R10	R15	R20	R25	R30
**3 × 30**	−0.2	−3.7	−3.7	−3.0	−3.3	−2.5	−1.7
**3 × 35**	−3.9	−6.0	−6.2	−5.9	−5.8	−3.6	−2.2
**3 × 40**	−5.1	−5.5	−5.9	−4.4	−2.9	−1.9	−0.4
**3 × 45**	−6.5	−6.6	−5.1	−2.2	−1.0	0.2	1.9
**3 × 50**	−6.9	−6.1	−4.1	−1.0	0.6	2.1	3.2
**3 × 55**	−6.6	−5.3	−2.2	1.3	3.4	5.3	5.5
**3 × 60**	−3.5	0.3	2.3	7.2	8.7	11.4	12.3
**6 × 30**	−0.7	−1.5	−1.4	−2.4	−1.4	−4.3	−2.3
**6 × 35**	−1.5	−2.0	−1.9	−1.8	−2.2	−1.1	−0.5
**6 × 40**	0.2	−1.3	−2.5	−3.0	−0.4	−0.8	0.0
**6 × 45**	−1.5	−3.0	−3.7	−3.2	0.3	−0.4	3.6
**6 × 50**	0.0	0.5	−1.9	−0.9	4.3	2.1	3.5
**6 × 55**	1.6	0.7	0.5	1.0	6.0	3.6	4.5
**6 × 60**	0.7	1.5	−0.1	1.7	7.2	6.1	8.6

## Data Availability

Data is contained within the article.
